# Strengthening Civil Registration Through Grassroots Health Institutions in India

**DOI:** 10.3390/ijerph23020257

**Published:** 2026-02-18

**Authors:** Sheetal Verma, Somnath Jana, Ritul Kamal, Laxmi Kant Dwivedi, Shiva S. Halli

**Affiliations:** 1 Department of Survey Research & Data Analytics, International Institute for Population Sciences (IIPS), Mumbai 400088, Maharashtra, India; sheetal.verma@nic.in (S.V.); somnath233@iipsindia.ac.in (S.J.); laxmikant@iipsindia.ac.in (L.K.D.); 2 Directorate of Census Operations, Lucknow 226024, Uttar Pradesh, India; ritulkamal.rgi@gov.in; 3 College of Community and Global Health, Institute for Global Public Health, Rady Faculty of Health Sciences, University of Manitoba, Winnipeg, MB R3E 0T6, Canada

**Keywords:** birth registration, institutional deliveries, civil registration system, legal identity

## Abstract

**Highlights:**

**Public health relevance—How does this work relate to a public health issue?**

- Civil registration is a foundational state function that ensures people’s legal identity, which is essential for children to access public health services, immunization programs, and rights protection.
- This study examines how the expansion of grassroots health infrastructure, specifically primary health centres (PHCs) and community health centres (CHCs), can be leveraged to achieve universal birth registration in India.

**Public health significance—Why is this work of significance to public health?**

- This study provides nationally representative evidence showing that births occurring in PHCs/CHCs have significantly higher odds of registration compared to private facilities.
- The findings highlight the role of decentralized public health infrastructure in reducing registration gaps among rural and socio-economically disadvantaged populations.

**Public health implications—What are the key implications or messages for practitioners, policy makers and/or researchers in public health?**

- Integrating civil registration functions within primary healthcare facilities can accelerate progress toward universal legal identity and SDG.
- Future policy should focus on deepening the digital integration between health service touchpoints and civil registration systems to reduce the administrative burden on frontline health workers while ensuring no child remains unregistered.

**Abstract:**

Civil registration of births and deaths underpins people’s legal identity, access to essential services, and evidence-based policy. Over the last two decades, the expansion of the National Health Mission (NHM) and the dramatic increase in institutional deliveries have created new opportunities to link maternal healthcare with critical event documentation. Primary health centres (PHCs) and community health centres (CHCs), which are frequently the initial point of contact for rural households, are emerging as important places for birthing and registration. Despite their expanding importance, the particular role of these grassroots facilities in birth registration results has not been thoroughly investigated. This study addresses that gap by assessing their role in increasing registration coverage among children under the age of five. We analyzed nationally representative data from the National Family Health Survey rounds 4 (2015–2016) and 5 (2019–2021). This study focused on children under five, examining the association between place of delivery and registration status. Descriptive analysis and multivariable logistic regression estimated the odds of registration across delivery settings. Pooled data from both survey rounds captured temporal shifts, and predicted probabilities were calculated for institutional deliveries, adjusting for socio-demographic covariates. The proportion of institutional births occurring in PHCs and CHCs rose from 30.5% to 34.7% between the two survey rounds. Registration among children delivered in these facilities increased from 80.8% to 90.2%, the highest gain among all delivery settings. Regression analysis showed that births in PHCs/CHCs were associated with 38% higher odds of being registered compared to private facilities. States designating PHCs and CHCs as official registrars, such as Delhi, Rajasthan, and Uttar Pradesh, reported the greatest improvements. Lower-level government health facilities are not only advancing safe delivery but also acting as pivotal nodes for civil registration. Their dual function creates a scalable model for integrating healthcare with legal identity creation, supporting equity and accelerating progress toward Sustainable Development Goal 16.9.

## 1. Introduction

Civil registration of vital events such as births and deaths is a foundational state function, essential for conferring legal identity on people, enabling access to public services, ensuring rights protection, and supporting evidence-based policy and planning [1,2,3]. In this context, Sustainable Development Goal (SDG) 16.9, which mandates legal identity for all, including universal birth registration by 2030, represents a global commitment to inclusive and accountable governance [4,5]. India, home to over a sixth of the world’s population, has made significant strides toward achieving this goal by integrating civil registration processes within the expanding framework of its public health infrastructure [3,6]. The National Health Mission (NHM), launched in 2005, has played a pivotal role in enhancing India’s health system by committing substantial investments in health infrastructure and service delivery, particularly in rural and underserved regions [7,8,9]. The NHM significantly contributed to the increase in institutional deliveries, which has substantially improved maternal and child health outcomes [8]. Concurrently, the institutionalization of births has facilitated the integration of birth registration into facility-based delivery protocols, markedly increasing the likelihood of registration, particularly among vulnerable socio-economic groups [3].

Lower-level government health facilities, such as primary health centres (PHCs) and community health centres (CHCs), being easily accessible to the rural populations makes them the first point of contact for all healthcare services and the primary choice for institutional births in various states of India [10,11,12]. The significant proliferation of PHCs and CHCs has played a pivotal role in the process. PHCs offer round the clock maternal care, while CHCs act as referral hubs for handling complicated deliveries. Between 2005 and 2021, PHCs and CHCs grew by approximately 8% and 60%, respectively [13]. Most importantly, in many states across the country, these health facilities either function directly as “Registrars” of births and deaths under the Registration of Births and Deaths Act, 1969 (2023) or act as critical informants to formal civil registration authorities [14,15].

The dual function of healthcare and civil registration ensures that childbirths occurring within these government-supported facilities are more systematically documented and officially registered. This shift also reflects growing policy recognition of the need to co-locate health service delivery and civil registration functions [3,16,17]. By ensuring that each institutional delivery becomes an opportunity for legal identity creation, PHCs and CHCs are playing an increasingly strategic role in advancing civil registration coverage [3,18,19]. Their direct access to timely and accurate information on births positions them uniquely to support real-time registration, minimize delays, and reduce underreporting, challenges that have long plagued India’s Civil Registration System.

The linkage between institutional deliveries and birth registration outcomes is supported by recent empirical evidence. According to the National Family Health Survey (NFHS)-5 (2019–21), 92% of births in India now occur in institutions, up from 86% in NFHS-4 (2015–16) [20,21]. Over the same period, birth registration coverage among children under age five increased from 80% to 89%, suggesting a strong positive correlation between increased institutional births and improved registration rates [20,21,22]. While these figures reflect national progress, they also obscure important sub-national dynamics, particularly the role of lower-level government health facilities such as PHCs and CHCs, which remain underexamined in quantitative terms. Although the existing literature acknowledges the general impact of institutional deliveries on registration across diverse regions, including India [3,23,24,25,26,27], there is limited disaggregated analysis of the differential contributions made by various categories of health facilities, especially those operating at the grassroots level and serving the most underserved populations.

Existing studies have largely documented the association between institutional deliveries and birth registration using descriptive comparisons or single survey rounds, offering limited insight into how specific types of facilities contribute to registration outcomes. In particular, the role of lower-level public health facilities such as PHCs and CHCs has remained analytically underexplored, despite their growing prominence in India’s maternal health system. By treating institutional delivery as a uniform category, prior research has often overlooked variations in administrative integration and registration practices across facility types. This study examines their effects by explicitly separating PHCs and CHCs from institutional delivery settings, using pooled nationally representative data from NFHS-4 (2015–16) and NFHS-5 (2019–21). 

This study seeks to fill that empirical gap by leveraging nationally representative data from NFHS-4 and NFHS-5 to rigorously examine the relationship between births occurring in PHCs and CHCs and the likelihood of subsequent official registration. It aims to isolate the specific contributions of these lower-tier government facilities, not just as sites of delivery but also as emerging nodes in the civil registration architecture. By doing so, this study sheds light on how India’s ongoing investments in health infrastructure are yielding dual dividends, improving health outcomes while also facilitating the realization of universal legal identity. The findings have important implications for the design of future health and administrative reforms, highlighting the potential for more integrated service delivery models that align civil registration with health system touchpoints. In doing so, this research contributes to a more granular understanding of the pathways through which legal identity is created and sustained in India, offering a data-driven foundation for more targeted and effective policy interventions going forward.

## 2. Materials and Methods

This study analyses birth registration trends in lower-level government health facilities using nationally representative data from NFHS-4 (2015–16) and NFHS-5 (2019–21). The NFHS provides standardized, district-level data on delivery location and registration outcomes. NFHS-5, carried out in two phases, covered all 707 districts across 28 states and eight union territories during June 2019 to April 2021, while NFHS-4 data collection spanned January 2015 to December 2016. NFHS-4 collected data from 601,509 households, 699,696 women, and 112,122 men, whereas NFHS-5 surveyed 636,699 households, 724,115 women, and 101,839 men [20,21].

The International Institute for Population Sciences (IIPS) conducted NFHS-4 in 2015–16 and NFH5 in 2019–21, providing vital health and family welfare data. The survey methodology, including detailed information on sampling, is available in the NFHS-4 [20] and NFHS-5 [21] reports.

### 2.1. Study Variables

The primary outcome variable in this analysis is birth registration. The NFHS collects de jure data on the number of children under five whose births have been registered with the civil authority. Specifically, NFHS-4 and NFHS-5 include the question: “Does a child have a birth certificate or has the child’s birth ever been registered by the civil authority?” Responses are categorized into four groups: “neither certificate nor registered,” “has certificate,” “registered,” and “don’t know.” For this study, the categories “has certificate” and “registered” have been combined, as the focus is on capturing whether the birth event was officially recorded.

In addition to birth registration, the place of delivery (recoded as “Home”, “Government facility”, “Private facility”, “CHC/PHC” and “Others”) has been considered in the analysis to study the role of lower-level public health facilities (CHC/PHC) in improving birth registration in India. Deliveries occurring in government facilities, private facilities and CHC/PHC have been considered “Institutional deliveries” in the analysis. Although PHCs and CHCs are administratively part of the public sector, they were analytically separated from higher-level government facilities to isolate their distinct role in birth registration; mutually exclusive NFHS delivery codes were used to avoid overlap across categories.

### 2.2. Statistical Analysis

Descriptive statistics were used to summarize the demographic and socio-economic characteristics of the study sample. This analysis used pooled data from two NFHS rounds—NFHS-4 (2015–2016) and NFHS-5 (2019–21). Data from NFHS-4 (2015–16) and NFHS-5 (2019–21) were pooled by appending the two survey rounds, and a survey-round indicator was included in all the pooled models to account for temporal differences. All the variables were interacted with the survey period to capture temporal variation. Binary logistic regression was employed to examine birth registration among children aged 0–5, using pooled data from NFHS-4 and NFHS-5. The model estimates the probability of registration (a binary outcome) using the maximum likelihood, with the coefficients interpreted as odds ratios. Predicted probabilities were estimated for institutional deliveries only, excluding home births, to focus on facility-based registration processes; home births follow different registration pathways and are therefore not directly comparable. All the statistical analyses were conducted using STATA 18 [28], and the figures are presented using Datawrapper [29], facilitating comprehensive visualization of the data. All the results are reported at the 5% level of significance. All the analyses accounted for the complex sampling design of the NFHS by applying sampling weights and adjusting for clustering and stratification, ensuring nationally representative estimates.

## 3. Results

**Figure 1:**
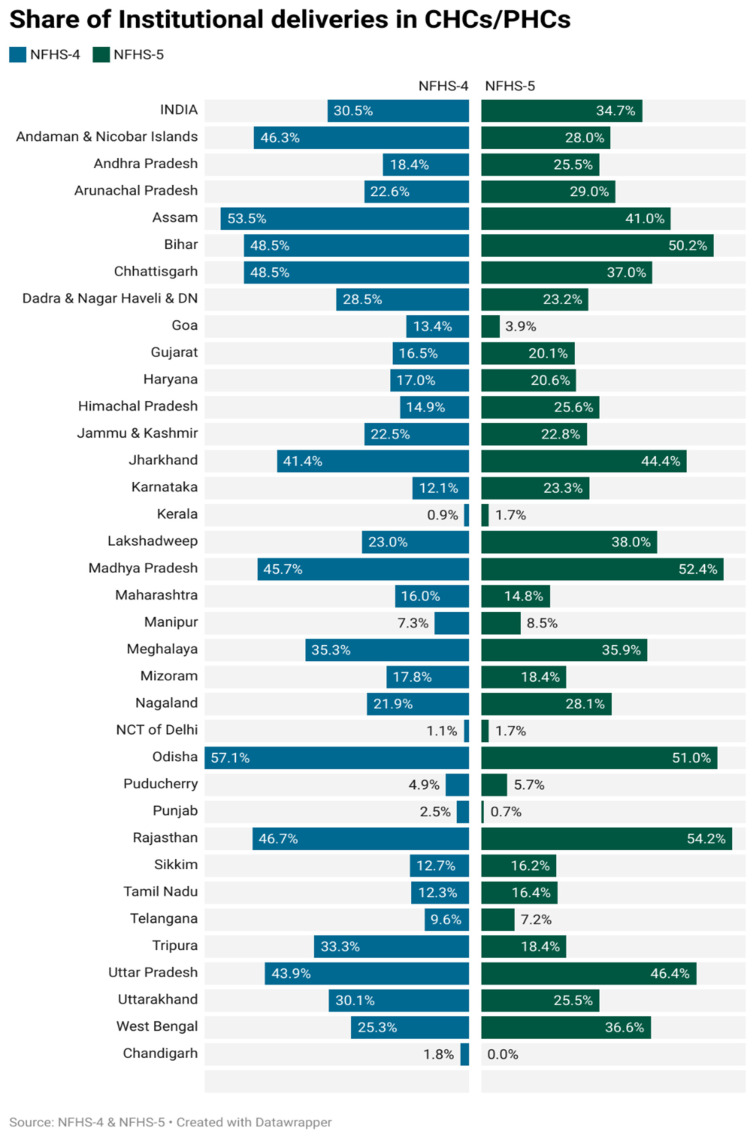
Share of institutional deliveries in CHCs/PHCs (NFHS-4 and NFHS-5). Note: PHC = primary health centre; CHC = community health centre. Institutional deliveries include government hospitals, private facilities, and PHCs/CHCs.

Figure 1 presents the share of institutional deliveries occurring in CHCs and PHCs across Indian states and union territories, as per NFHS-4 (2015–16) and NFHS-5 (2019–21). It highlights substantial inter-state variation in the share of institutional deliveries occurring in PHCs and CHCs, with several states showing marked increases over time, while a smaller number exhibit stagnation or decline. At the national level, the proportion of institutional deliveries in CHCs and PHCs increased from 30.5% in NFHS-4 to 34.7% in NFHS-5. Several states witnessed substantial improvements. Rajasthan recorded the highest increase, rising from 46.7% to 54.2%, followed by Madhya Pradesh (45.7% to 52.4%), Jharkhand (41.4% to 44.4%) and Uttar Pradesh (43.9% to 46.4%). Assam, although experiencing a decline (from 53.5% to 41.0%), remained among the states with relatively high CHC and PHC utilization. In contrast, certain states like Goa saw a significant drop from 13.4% to 3.9%, and Andaman and Nicobar Islands from 46.3% to 28.0%. Similar declines were witnessed in Dadra and Nagar Haveli (28.5% to 23.2%) and Punjab (2.5% to 0.7%).

**Figure 2:**
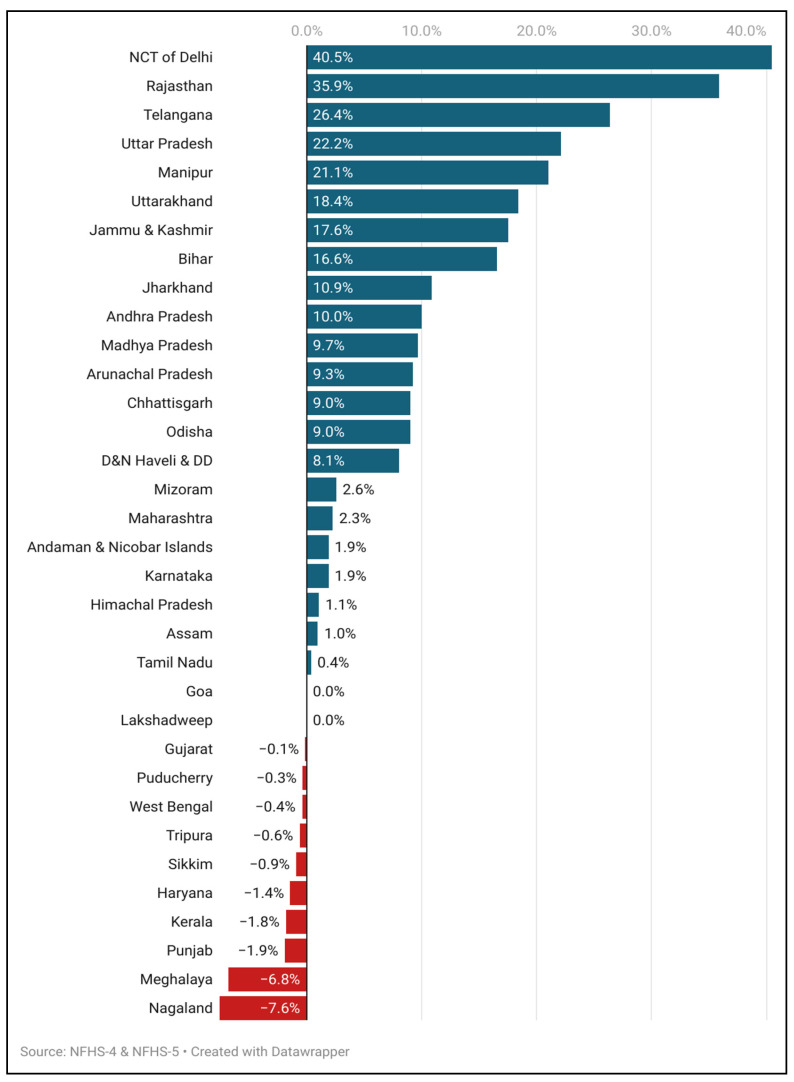
Relative change in birth registration in CHCs/PHCs (NFHS-4 and NFHS-5).

Figure 2 highlights the relative change in birth registration among institutional deliveries occurring in CHCs and PHCs, disaggregated by states and union territories. The most substantial increases were observed in the National Capital Territory (NCT) of Delhi (40.5%), Rajasthan (35.9%) and Telangana (26.4%). Other states with notable improvements included Uttar Pradesh (22.2%), Manipur (21.1%), Uttarakhand (18.4%), Jammu and Kashmir (17.6%), and Bihar (16.6%). States such as Jharkhand, Andhra Pradesh and Madhya Pradesh witnessed moderate improvements in birth registration among institutional deliveries occurring in CHCs and PHCs. In contrast, states like Nagaland (−7.6%), Meghalaya (−6.8%), Punjab (−1.9% and Kerala (−1.8%) witnessed declines in birth registration rates. Union territories of Lakshadweep and states like Goa reported no change.

Notably, the majority of the states and union territories, like the NCT of Delhi, Uttar Pradesh, Manipur, Uttarakhand and Bihar, which have witnessed the highest increase in the birth registration rate, have designated the CHCs and PHCs as “Registrars” for registering vital events, except for Telangana and Jammu and Kashmir, which is indicative of a strong potential link between institutional deliveries and universal birth registration (Figure 2).

**Figure 3:**
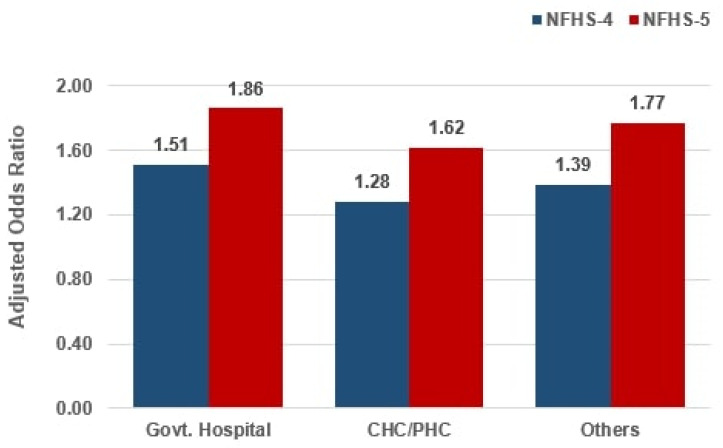
Comparison of the odds ratio of birth registration by place of delivery. Note: Sex, religion and caste of the head of household, wealth index, health insurance availed, household structure, education of father, education of mother, place of residence, media exposure and birth order were adjusted in the logistic regression model. PHC = primary health centre; CHC = community health centre. Institutional deliveries include government hospitals, private facilities, and PHCs/CHCs.

Figure 3 presents the results of the multivariable logistic regression analysis examining the association between place of delivery and birth registration. The model estimated adjusted odds ratios for birth registration among institutional deliveries, comparing data from NFHS-5 and NFHS-4, while controlling for covariates. The results of the multivariable logistic regression highlight that, after adjusting for covariates, births occurring in CHCs/PHCs were associated with an odds ratio of 1.62 in NFHS-5, as compared to private hospitals, which corresponds to ~27% increase in odds from NFHS-4 (Table S1). These findings underscore the pivotal role of public health facilities in facilitating higher birth registration coverage and advancing progress toward universal birth registration in India. These estimates should be interpreted as adjusted associations rather than causal effects.

**Figure 4:**
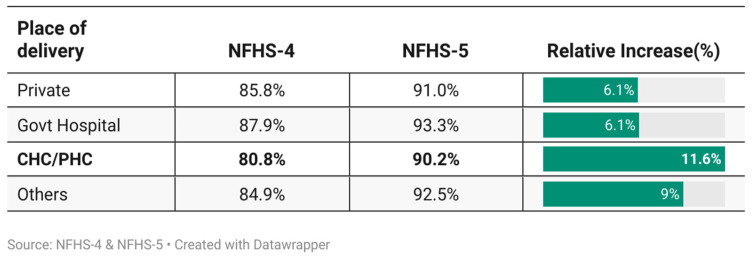
Predicted probabilities of birth registration by place of delivery. Note: Sex, religion and caste of the head of household, wealth index, health insurance availed, household structure, education of father, education of mother, place of residence, media exposure and birth order were adjusted in the logistic regression model. PHC = primary health centre; CHC = community health centre. Institutional deliveries include government hospitals, private facilities, and PHCs/CHCs.

Figure 4 shows the differences in the adjusted predicted probabilities of birth registration across delivery settings and survey rounds, highlighting the variation net of the observed characteristics. To assess the changes in birth registration by place of delivery between 2015 and 2021, we also conducted a binary logistic regression using pooled data from NFHS-4 and NFHS-5, controlling for relevant covariates. The resulting predicted probabilities illustrated variations in registration across different delivery locations. Figure 4 shows improved birth registration across all delivery settings from NFHS-4 to NFHS-5, with CHCs/PHCs seeing the largest rise from 80.8% to 90.2% (11.6% growth). Private and government hospitals showed smaller increases of 6% and 6.1%, respectively (Figure 4).

## 4. Discussion

Institutional deliveries in India have undergone a seismic transformation over the past two decades, fundamentally altering the country’s approach to maternal and child health as well as to vital event documentation. At the heart of this evolution is the dynamic convergence between expanded public healthcare infrastructure and the systematic registration of births, with PHCs and CHCs emerging as pivotal agents of both clinical care and civil registration.

From 2005–06 (NFHS-3) to 2019–21 (NFHS-5), India achieved a remarkable leap forward in institutional deliveries, propelling the rate from a modest 39% to an impressive 89% [10,21]. This 128% surge represents not just increased access to safe childbirth but a reorientation of the entire maternal healthcare landscape toward institutional care. Underpinning this transformation have been sustained investments, robust policy interventions like the National Health Mission (NHM) [9], and innovative demand-side schemes such as the Janani Suraksha Yojana (JSY) [30], which uses conditional cash transfers to incentivize facility-based births particularly among the poor and socially marginalized.

PHCs and CHCs, typically considered secondary or even peripheral-level providers, have quickly grown in both capacity and relevance. By the time NFHS-5 concluded in 2021, these lower-tier public facilities accounted for nearly 31% of all institutional births, signalling their ascending role as both clinical and administrative hubs [21]. Crucially, PHCs and CHCs contributed to approximately 62% of the growth in institutional deliveries over the last decade, a testament to their decentralizing influence and grassroots reach [21]. Rather than serving narrowly as clinical outlets, these centres have also become critical registration nodes for the civil registration of births [3,18].

The intrinsic link between the rise of institutional deliveries and the completeness of birth registration is now well established. As the proportion of women delivering in healthcare facilities climbed from 79% in NFHS-4 (2015–16) to 89% in NFHS-5 (2019–21), the proportion of children born in PHCs and CHCs who were registered also grew from 80% to 90% [20,21]. This near-parallel progression underscores a vital feedback loop: as more births occur within institutions, the administrative process for timely, complete birth registration becomes both natural and seamless. Here, the integration of civil registration directly into maternity care plays a transformative role, making it an in-built component rather than a burdensome afterthought.

The importance of facility choice is further substantiated by the multivariable logistic regression analyses, which indicate that children born in CHCs or PHCs had 38% higher odds of being registered compared to those born in private hospitals, with a comparable advantage seen in government-run hospitals (37% higher odds) (Table S1). This suggests that administrative and structural synergies between the public healthcare system and civil registration authorities are delivering tangible benefits, especially for the poorest and most remote families. These findings represent adjusted associations and should not be interpreted as evidence of a causal effect of place of delivery on birth registration.

The decentralization of maternal healthcare, driven by the expansion of PHCs and CHCs, has profound implications for equity. These centres are not only geographically closer to rural and underserved populations but are also embedded in the daily life and trust networks of local communities [31,32,33]. Their designation as “registrars” or key informants to the registrar further lowers the transaction costs, eliminating the need for families to visit distant government offices or navigate opaque bureaucracies for birth certificates [15]. Scheduled and repeated outreach often powered by frontline health workers such as Accredited Social Health Activists (ASHAs) and Auxiliary Nurse Midwives (ANMs)brings birth documentation within immediate reach for even the most marginalized, bridging gaps of geography, information, and social capital.

Technological interventions have further turbocharged this process. The advent of digital platforms like API Setu and DIGIT, as well as the roll-out of mobile civil registration apps, has allowed for the seamless, near-instant exchange of birth data between health, registration, and welfare agencies [34,35,36]. This connectivity ensures minimal redundancy, speeds up the issuance of birth certificates, which are mandatory for a host of services according to the 2023 RBD Amendment, and makes the process user-friendly, efficient, and transparent. Conditional welfare schemes (JSY, Pradhan Mantri Matru Vandana Yojana, and others) are now digitally linked to birth registration, so that legal identity becomes both a right and a gateway to essential services, fundamentally altering the incentive structure for institutional deliveries.

By acting as “one-stop shops” for both clinical care and legal documentation, PHCs and CHCs are driving a virtuous cycle: institutional delivery enables timely registration, which then leads to broader social and legal inclusion for the child. From access to healthcare and immunization to inclusion in systems of education and social protection, a registered identity is foundational to realizing the full benefits of citizenship. For millions of children, this dual intervention ensures not only their immediate survival and health but also long-term equity and mobility [3,18,24,37].

Despite the progress, integrating registration into health services strains frontline workers like ANMs and ASHAs, who already juggle maternal care, immunization, nutrition, family planning, and now civil documentation often without sufficient training, support, or compensation, which risks undermining core healthcare delivery. Sustaining gains requires continuous capacity-building, dedicated documentation staff, and user-friendly digital tools that reduce rather than add to workloads. At the systemic level, the lack of real-time, granular integration between health events and civil registration remains a critical gap. While the NFHS surveys offer useful snapshots, linking facility-level reporting with registration data and further connecting digital health records to the Civil Registration System would minimise delays, ease administrative burdens and enable proactive outreach to unregistered children.

Importantly, this synthesis of healthcare and civil registration is especially critical for India’s commitment to Sustainable Development Goal (SDG) 16.9: “legal identity for all.” The health sector, given its routine contact with every birth, stillbirth, and neonatal death, occupies a uniquely strategic position. Leveraging this position to ensure that no birth goes unregistered is foundational for governance, social protection, and the realization of basic human rights. As India’s maternal and child health, immunization, and nutrition programs expand, so too does the opportunity to institutionalize registration as a natural extension of care.

The ascendancy of PHCs and CHCs as “registration nodes” has particular resonance for the most marginalized populations, those for whom distance, administrative complexity, and social stigma have long impeded access to identity and entitlements. For these families, the local public health centre is often their most consistent and sometimes only interface with the state. Making birth registration both convenient and automatic at this first point of contact fundamentally transforms the experience, ensuring that children begin their lives with the recognition, rights, and protections owed to them.

This study has some limitations. The analysis is based on cross-sectional, observational survey data, which limits causal inference. Birth registration status is self-reported in the NFHS and may be subject to recall or reporting bias. While the use of pooled nationally representative data strengthens generalizability, unobserved institutional and state-level factors may continue to influence registration outcomes.

In conclusion, India’s pursuit of near-universal institutional births and comprehensive birth registration is increasingly anchored in the decentralized strength of its PHCs, CHCs, and related grassroots health infrastructure. By integrating the registration process directly into the routine of childbirth and maternity care, supported by digital platforms, welfare incentives, and frontline outreach, the country is not only improving maternal and child health outcomes but also extending the reach of citizenship, equity, and social protection. Future progress will depend on sustaining this integration, addressing operational challenges, and deepening the real-time connection between health events and civil documentation. As these lower-level public facilities continue their twin ascent as providers of both safe childbirth and legal identity, they are laying the foundation for a more equitable and inclusive society, one birth and one certificate at a time.

## Data Availability

The data is available online on the website and can be downloaded. Data used in this study is publicly available and can be accessed from the DHS program website (https://dhsprogram.com/methodology/survey/survey-display-355.cfm, accessed on 20 October 2024).

## Supplementary Materials

The following supporting information can be downloaded at https://www.mdpi.com/article/10.3390/ijerph23020257/s1, Table S1: Multivariable logistic regression results.
