# Supplementary material for: Strengthening Civil Registration Through Grassroots Health Institutions in India
Source: Int J Environ Res Public Health. 2026 Feb 18;23(2):257. doi: 10.3390/ijerph23020257 (PMC12940596; doi:10.3390/ijerph23020257)
Supplement: Supplementary file 1 [file ijerph-23-00257-s001.zip › ijerph-4088870-supplementary.pdf]

Supplementary Table S1: Multivariable logistic regression results

Birth registered	Odds Ratio (95% CI)	
	NFHS-5	NFHS-4
**Place of delivery**		
Private	1.00 (ref)	1.00 (ref)
Govt. Hospital	1.86 (1.78 – 1.96)	1.51 (1.47 – 1.58)
CHC/PHC	1.62 (1.49 – 1.63)	1.28 (1.18 – 1.27)
Others	1.77 (1.6- - 1.89)	1.39 (1.29 – 1.45)
**Sex of Head of Household**		
Male	1.00 (ref)	1.00 (ref)
Female	0.86 (0.82 – 0.90)	0.99 (0.95 – 1.03)
**Religion of Head of Household**		
Hindu	1.00 (ref)	1.00 (ref)
Muslim	1.01 (0.96 – 1.07	0.86 (0.82 – 0.90)
Others	0.84 (0.79 – 0.90)	1.14 (1.08 – 1.21)
**Category of Head of Household**		
Scheduled Caste	1.00 (ref)	1.00 (ref)
Scheduled Tribe	1.14 (1.08 – 1.20)	0.98 (0.93 – 1.02)
Others	0.93 (0.89 – 0.97)	0.89 (0.86 – 0.92)
**Wealth Index**		
Poorest	1.00 (ref)	1.00 (ref)
Poorer	1.41 (1.34 – 1.47)	1.33 (1.29 – 1.38)
Middle	1.88 (1.78 – 1.98)	1.79 (1.71 – 1.86)
Richer	2.48 (2.33 – 2.63)	2.37 (2.25 – 2.49)
Richest	3.78 (3.49 – 4.09)	3.71
**Health Insurance**		
No	1.00 (ref)	1.00 (ref)
Yes	1.47 (1.42 – 1.53)	1.62 (1.56 – 1.68)
**Household Structure**		
Nuclear	1.00 (ref)	1.00 (ref)
Non-nuclear	1.04 (1.01 – 1.08)	0.93 (0.91 – 0.96)
**Education of mother**		
No education	1.00 (ref)	1.00 (ref)
Primary	1.46 (1.38 – 1.54)	1.55 (1.48 – 1.61)
Secondary	1.96 (1.88 – 2.05)	1.99 (1.92 – 2.06)
Higher	1.99 (1.85 – 2.13)	1.98 (1.86 – 2.11)
